# Pharmacokinetic Profile of Matrine in Pigs Following Intravenous and Oral Administration

**DOI:** 10.3390/vetsci13070652

**Published:** 2026-07-03

**Authors:** Jianzhong Wang, Hang Yan, Jing Liu, Rui Zhou, Wei Yin, Jia Zhong, Panpan Sun, Na Sun, Zhenbiao Zhang, Yaogui Sun, Huizhen Yang, Kuohai Fan, Hongquan Li

**Affiliations:** Shanxi Key Laboratory for Modernization of TCVM, College of Veterinary Medicine, Shanxi Agricultural University, Taigu 030801, China

**Keywords:** matrine, pharmacokinetics, pig, UPLC–MS/MS

## Abstract

The search for effective alternatives or adjuncts to conventional anti-infective strategies in food animals has increased interest in plant-derived bioactive compounds. Matrine, a quinolizidine alkaloid derived from *Sophora flavescens*, has attracted considerable attention because of its broad bioactivities and potential veterinary relevance. Recent oral pharmacokinetic and intestinal-lumen PBPK studies in pigs have begun to emerge; however, currently available swine data remain largely confined to oral exposure-oriented experiments and do not permit direct route comparison or estimation of absolute oral bioavailability. In the present study, the plasma pharmacokinetics of matrine in pigs were evaluated after single-dose intravenous and oral administration. Twelve healthy piglets received matrine at 8 mg/kg body weight via either the intravenous or oral route, and plasma concentrations were quantified using a validated UPLC–MS/MS method. Pharmacokinetic parameters were estimated by non-compartmental analysis. Compared with intravenous administration, oral dosing produced lower observed exposure based on ${AUC}_{0-t}$ and a lower peak plasma concentration, with ${AUC}_{0-t}$ and $C_{\max}$ values of 418.94 ± 75.52 h·ng/mL and 66.24 ± 8.44 ng/mL, respectively, versus 558.01 ± 59.57 h·ng/mL and 224.64 ± 20.94 ng/mL after intravenous administration. Oral administration was associated with a $T_{\max}$ of 2.49 ± 0.02 h and a longer apparent terminal half-life and mean residence time than intravenous dosing. These findings provide pharmacokinetic evidence to support future route selection, dosing-regimen design, and PK/PD-based dose optimization of matrine in pigs.

## Simple Summary

Matrine is an alkaloid obtained mainly from *Sophora flavescens* and has been investigated for anti-inflammatory and antiviral activities. Pharmacokinetic information for matrine in pigs is still limited, which makes evidence-based veterinary use difficult. This study examined the absorption, distribution, and elimination of matrine after a single oral or intravenous dose in pigs. Oral dosing produced a lower peak plasma concentration, a delayed time to peak concentration, and a longer apparent half-life than intravenous dosing. These data provide a pharmacokinetic foundation for future dosing-regimen design and for the further evaluation of matrine in swine medicine.

## 1. Introduction

**Figure 1:**
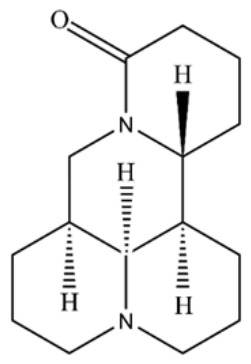
Chemical structure of matrine.

Matrine (MT), a tetracyclic quinolizidine alkaloid derived primarily from Sophora flavescens Ait [1,2], has been reported to possess a broad spectrum of pharmacological activities, including antiviral [3,4], anti-inflammatory [5,6], antitumor [7,8], and hepatoprotective effects [9]. The chemical structure of matrine is shown in Figure 1. Recent studies have shown that matrine exerts these effects through modulation of multiple signaling pathways, including the TLR4/NF-κB and MAPK pathways, and through regulation of oxidative stress and immune responses [4,5,6]. In addition, matrine has demonstrated activity against several swine-related viral pathogens, highlighting its potential relevance in animal health applications [3,4]. Pigs represent an important livestock species in modern animal production and are a major target species for veterinary therapeutic intervention. Therefore, species-specific pharmacokinetic information on matrine in pigs is needed to support its evidence-based evaluation and future application in swine medicine.

From a clinical pharmacology perspective, the pharmacokinetic behavior of a drug is a critical determinant of its efficacy and safety. Drug exposure is influenced by multiple factors, including the route of administration, species-specific physiological characteristics, formulation properties, and biotransformation capacity [10]. These factors may substantially alter the absorption, distribution, metabolism, and excretion of a compound, thereby affecting its therapeutic performance in vivo. Accordingly, pharmacokinetic characterization is an essential prerequisite for the rational design of dosage regimens and for the subsequent establishment of pharmacokinetic–pharmacodynamic (PK/PD) relationships.

Previous pharmacokinetic studies on matrine have mainly been conducted in rodents [11], rabbits [12], and dogs [13,14,15]. These investigations have shown that the disposition of matrine can vary substantially across species and formulations. For example, oral bioavailability in rats has been reported to be relatively low [16,17], whereas herbal co-administration may alter the systemic exposure of matrine [15,18]. Recently, a pharmacokinetic study in pigs investigated the pharmacokinetics of matrine in pigs following oral gavage administration of matrine at 20 mg/kg, either alone or in combination with amoxicillin, and reported rapid absorption and elimination of the compound [19]. Subsequently, another study characterized the kinetic behavior of matrine in the intestinal lumen after oral administration and developed a physiologically based pharmacokinetic (PBPK) model to describe its intestinal disposition in pigs. However, these studies were limited to oral administration and did not permit direct comparison between intravenous and oral routes or estimation of absolute oral bioavailability. Therefore, a route-comparative pharmacokinetic evaluation remains necessary to better characterize the systemic disposition of matrine in swine [20].

The present research was conducted to characterize the plasma pharmacokinetics of matrine in pigs following intravenous and oral administration using a validated UPLC–MS/MS method. Although recent studies have described the pharmacokinetics of matrine following oral administration and have explored its intestinal disposition in pigs, these investigations could not determine absolute oral bioavailability or directly compare route-dependent pharmacokinetic behavior because an intravenous comparator was not included. Oral pharmacokinetic studies provide information on absorption characteristics and systemic exposure under clinically relevant conditions, whereas intravenous administration allows characterization of drug disposition and elimination independent of the absorption process. Therefore, the inclusion of an intravenous treatment group enabled a more comprehensive evaluation of matrine disposition in pigs. The resulting pharmacokinetic information may provide a basis for future route selection, dose optimization, and PK/PD-based evaluation of matrine in swine medicine.

## 2. Materials and Methods

### 2.1. Chemicals and Reagents

Matrine reference standard (purity, 98.7%; batch no. 110708-200505) was obtained from the National Institutes for Food and Drug Control (Beijing, China). Acetonitrile (HPLC grade) was purchased from Shanghai Macklin Biochemical Technology Co., Ltd (Shanghai, China). Formic acid (LC–MS grade) was supplied by Thermo Fisher Scientific. (Waltham, MA, USA) Phosphoric acid (analytical grade) was purchased from Tianjin Beichen Fangzheng Reagent Factory (Tianjin, China), and perchloric acid (guaranteed reagent grade) was obtained from Sinopharm Chemical Reagent Co., Ltd. (Shanghai, China).

### 2.2. Animals

Twelve clinically healthy crossbred piglets were used in this experiment (8–9 weeks old; body weight 20 ± 2.5 kg; six males and six females). Before dosing, animals were acclimated for 7 days in individual pens under routine husbandry conditions. Room temperature was maintained at 18–28 °C, relative humidity at 40–60%, and lighting on a 12 h light/12 h dark cycle. Clinical health was confirmed before drug administration. The protocol was approved by the Institutional Animal Care and Use Committee of Shanxi Agricultural University (approval no. SXAU-EAW-2024P.AF.006023232).

### 2.3. Experimental Design

The animals were randomly assigned to two groups according to the route of administration: an oral administration group (*n* = 6 per group). and an intravenous administration group. Each group contained three males and three females. Prior to dosing, pigs were fasted for 12 h with free access to water. Matrine was dissolved in a small volume of DMSO and then diluted with sterile 0.9% sodium chloride solution immediately before administration. Matrine was administered as a single dose of 8 mg/kg body weight to both groups. The dose was selected based on preliminary pharmacodynamic studies conducted by our research group, in which 8 mg/kg was found to provide an acceptable biological response and safety profile in pigs. Furthermore, this dose was expected to produce plasma concentrations that could be reliably quantified over the planned sampling period.

For the oral group, blood samples (approximately 4 mL) were collected from the anterior vena cava before dosing and at 0.5, 1, 1.5, 2, 2.5, 3, 4, 6, 8, 12, 16, and 24 h after administration. For the intravenous group, blood samples were collected at 0, 0.08, 0.25, 0.5, 1, 2, 3, 4, 6, 8, 12, 16, and 24 h after dosing. Samples were collected into heparinized tubes, centrifuged at 3000 rpm for 10 min at 4 °C, and the plasma was separated and stored at −20 °C until analysis.

### 2.4. Sample Preparation

Plasma samples were thawed at room temperature and processed before injection. For each sample, 1 mL plasma was combined with 3 mL acetonitrile and vortexed for 2 min. After centrifugation at 10,000 rpm for 10 min at 4 °C, the supernatant was mixed with perchloric acid at a 1:0.6 volume ratio, vortexed again for 2 min, and centrifuged at 12,000 rpm for 5 min. The upper acetonitrile phase was transferred and evaporated under nitrogen in a 35 °C water bath. The dry residue was reconstituted in 200 μL of water/acetonitrile (1:1, *v*/*v*), each containing 0.1% formic acid, centrifuged at 16,000 rpm for 15 min at 4 °C, and filtered through a 0.22 μm membrane prior to UPLC-MS/MS analysis.

### 2.5. UPLC–MS/MS Conditions

Matrine in plasma was quantified by UPLC-MS/MS using positive electrospray ionization and multiple reaction monitoring. Separation was performed on an Accucore C18 column (2.1 mm × 50 mm, 1.9 μm) held at 40 °C. Mobile phase A was water with 0.1% formic acid, and mobile phase B was acetonitrile with 0.1% formic acid. The flow rate was 0.3 mL/min, and 5 μL was injected. The gradient was programmed as follows: 0–2 min, 1% B; 2–4 min, 90% B; 4–5 min, 90% B; 5–5.5 min, 1% B; and 5.5–6 min, 1% B.

### 2.6. Calibration Standards and Quality Control Samples

A 1 mg/mL matrine stock solution was prepared in acetonitrile and kept at 4 °C. Working solutions were produced by serial dilution in acetonitrile at 100, 200, 500, 1000, 2000, 5000, and 10,000 ng/mL. Quality-control samples were generated by adding the appropriate working solutions to blank pig plasma to obtain final concentrations of 10, 100, and 500 ng/mL.

### 2.7. Method Validation

The analytical procedure was established with reference to previously reported LC–MS/MS and UPLC–MS/MS methods for matrine determination in plasma samples [15,16,19,21] and was further optimized for porcine plasma in the present study. Validation followed the general principles of ICH M10 bioanalytical method validation, focusing on selectivity, calibration performance, accuracy, precision, recovery, and stability. The analytical method showed good linearity over the concentration range of 10–1000 ng/mL, with a calibration equation of *Y* = 5.925 × $10^{3}$*X* + 1.925 × $10^{5}$ and an $R^{2}$ value of 0.9993. The retention time of matrine was 0.97 min, and no endogenous interference was detected in blank plasma samples at this retention time. Mean recovery ranged from 86.27% to 118.37% across the tested quality-control levels. Intra-day precision ranged from 1.5% to 14.7%, whereas inter-day precision ranged from 2.3% to 6.8%. The recovery values reported here should be interpreted as apparent extraction recovery. Recovery values slightly exceeding 100% may reflect matrix-related ion enhancement or minor variation between extracted and post-extraction spiked samples rather than true over-recovery of the analyte. The wider intra-day precision range was mainly associated with concentration-dependent variability, particularly at lower quality-control levels. However, the observed accuracy and precision remained within acceptable limits for bioanalytical method validation, indicating that the method was suitable for pharmacokinetic analysis of matrine in porcine plasma. Stability testing demonstrated that matrine remained stable in plasma at room temperature, after 24 h storage at room temperature, and after three freeze–thaw cycles. Collectively, these results indicate that the assay was suitable for the intended pharmacokinetic application in porcine plasma.

### 2.8. Data Analysis

Plasma concentration–time data obtained after single oral and intravenous administration of matrine were analyzed by non-compartmental analysis using PKanalixTM 2024R1 (Lixoft SAS, Antony, France). The observed values of $C_{\max}$ and $T_{\max}$ were obtained directly from the concentration–time profiles. Other pharmacokinetic parameters, including AUC, MRT, terminal elimination half-life, clearance, and apparent volume of distribution, were estimated using standard non-compartmental procedures. Because extravascular pharmacokinetic parameters are influenced by systemic bioavailability, the oral clearance and volume of distribution are reported as CL/F and Vz/F, respectively, rather than as physiologic intravenous parameters. Absolute oral bioavailability was calculated according to the following equation:

$$F(\%)=\frac{{\mathrm{AUC}}_{po}}{{\mathrm{AUC}}_{iv}}\times \frac{{\mathrm{Dose}}_{iv}}{{\mathrm{Dose}}_{po}}\times 100$$

All pharmacokinetic data are presented as mean ± standard deviation (SD). Given the exploratory nature of this study and the limited sample size, the results are interpreted primarily in a descriptive pharmacokinetic framework. Pharmacokinetic parameters were summarized descriptively as mean ± SD. Because the study was designed as an exploratory route-comparative pharmacokinetic investigation and several parameters after oral administration represent apparent values influenced by bioavailability, no formal inferential statistical comparison between administration routes was performed.

## 3. Results

**Figure 2:**
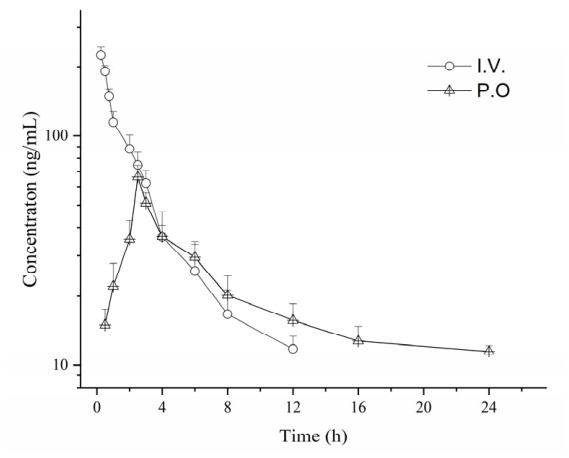
Mean plasma concentration–time profiles of matrine in pigs following a single intravenous or oral administration at 8 mg/kg body weight, presented on a semi-logarithmic scale.

No obvious adverse clinical signs, including abnormal behavior, reduced appetite, vomiting, diarrhea, or injection-site reactions, were observed during the 24 h observation period after either intravenous or oral administration. The mean plasma concentration–time profiles are shown in Figure 2, which is presented on a semi-logarithmic scale to facilitate visualization of the terminal phase, and the corresponding pharmacokinetic parameters are summarized in Table 1. Because the AUC extrapolated after oral administration exceeded 20%, terminal-phase-dependent oral pharmacokinetic parameters were not reported.

After intravenous administration, plasma matrine concentrations were highest at the first sampling time and then declined over the sampling period. The ${AUC}_{0-t}$, ${AUC}_{0-\infty }$, $C_{max}$, terminal half-life, and ${MRT}_{0-t}$ after intravenous administration were 558.01 ± 59.57 h·ng/mL, 634.58 ± 67.91 h·ng/mL, 224.64 ± 20.94 ng/mL, 4.09 ± 0.80 h, and 2.72 ± 0.58 h, respectively. The AUC extrapolated after intravenous administration was 11.98 ± 4.19%, indicating that the observed sampling period adequately characterized most of the systemic exposure.

After oral administration, matrine reached a peak plasma concentration of 66.24 ± 8.44 ng/mL at 2.49 ± 0.02 h. The ${AUC}_{0-t}$ and ${AUC}_{0-\infty }$ were 418.94 ± 75.52 h·ng/mL and 698.31 ± 141.19 h·ng/mL, respectively. The apparent terminal half-life and MRT_0–t_ after oral administration were 16.40 ± 7.70 h and 7.36 ± 1.70 h, respectively. The AUC extrapolated after oral administration was 38.24 ± 14.40%, indicating that the terminal phase after oral dosing was not sufficiently characterized. Therefore, ${AUC}_{0-\infty }$, λz-derived T_1/2_, CL/F, Vz/F, and absolute oral bioavailability after oral administration were not reported or interpreted.

Descriptive comparison of the route-dependent pharmacokinetic parameters showed that oral administration resulted in lower systemic exposure and peak plasma concentration than intravenous administration. Oral ${AUC}_{0-t}$ was approximately 75.1% of the intravenous value, whereas oral $C_{max}$ was approximately 29.5% of the intravenous value. Because terminal-phase-dependent oral parameters were considered unreliable, comparisons involving oral CL/F, Vz/F, T_1/2_, or absolute bioavailability were not performed. These comparisons were descriptive, and no formal statistical testing between administration routes was performed.

## 4. Discussion

The present study characterized the plasma pharmacokinetics of matrine in pigs following single-dose intravenous and oral administration. The results demonstrated clear route-dependent differences in systemic exposure and disposition, and they provide a preliminary estimate of the absolute oral bioavailability of matrine in this species. This study therefore extends the currently available swine literature, which has primarily focused on oral exposure-oriented experimental designs, by incorporating an intravenous arm and enabling route-comparative interpretation of matrine disposition in pigs [19,22]. Because the AUC extrapolated after oral administration exceeded 20%, terminal-phase-dependent oral pharmacokinetic parameters and absolute oral bioavailability were not reported or interpreted.

The present results are generally consistent with previous swine studies but also provide additional information. A pig gavage study of matrine alone or combined with amoxicillin reported rapid absorption and elimination, and a PBPK-oriented study showed that intestinal-lumen concentrations peaked at about 2 h after oral dosing [19,20]. The oral $T_{max}$ observed in the present study was 2.49 h, which is broadly consistent with these previous observations. However, direct comparison of AUC, $C_{max}$, and apparent clearance among studies should be made cautiously because of differences in dose, formulation, biological matrix, sampling design, and analytical methods. Unlike previous swine studies, the present investigation included an intravenous comparator, allowing preliminary estimation of absolute oral bioavailability and clearer interpretation of route-dependent pharmacokinetic behavior. Although the intravenous comparator improved route-based interpretation, the oral terminal phase was not sufficiently characterized to support reporting of CL/F, Vz/F, T1/2, or F.

Compared with studies in other animal species, the pharmacokinetic profile of matrine in pigs showed several distinct features. In rats, oral matrine exposure has generally been reported to be limited, with an absolute bioavailability of approximately 17.1% in one study [16], whereas another rat study also indicated incomplete systemic availability after oral dosing [17,23]. By contrast, the estimated oral bioavailability in pigs in the present study was 75.08% based on ${AUC}_{0-t}$, suggesting that matrine may have relatively favorable oral systemic availability in this species. In dogs, oral administration of matrine-containing preparations has been associated with rapid absorption and relatively high systemic exposure, indicating that both species and formulation can substantially influence matrine disposition [15]. In rabbits, matrine-containing preparations showed rapid distribution and elimination after injection [12], which is broadly consistent with the relatively rapid decline in plasma concentrations observed after intravenous administration in pigs. These interspecies differences may be related to variation in gastrointestinal transit, luminal pH, intestinal permeability, transporter activity, gut microbial metabolism, hepatic metabolic capacity, and cytochrome P450 expression and function [24,25]. Because terminal-phase-dependent oral parameters were not reliable in the present study, cross-species comparison was restricted mainly to observed exposure and peak-time characteristics rather than absolute bioavailability or oral clearance.

A major finding of the present study was that oral administration produced lower ${AUC}_{0-t}$ and $C_{max}$ values than intravenous administration at the same nominal dose. Quantitatively, oral ${AUC}_{0-t}$ was approximately 75.1% of the intravenous value, corresponding to a reduction of about 24.9%, whereas oral $C_{max}$ was approximately 29.5% of the intravenous value, corresponding to a reduction of about 70.5%. These descriptive comparisons indicate that the route of administration markedly influenced the observed exposure over the sampling interval and the peak plasma concentration. Mechanistically, the lower $C_{max}$ and delayed $T_{max}$ after oral administration likely reflect the time required for gastrointestinal dissolution, intestinal absorption, and transfer into the systemic circulation, whereas the lower oral AUC_0–t_ is consistent with incomplete absorption and/or presystemic loss [26,27]. However, because the terminal phase after oral administration was not sufficiently characterized, the present data should not be used to infer oral clearance, volume of distribution, terminal half-life, or absolute bioavailability.

The relatively high oral bioavailability should be interpreted cautiously. In this study, bioavailability was estimated using ${AUC}_{0-t}$ rather than ${AUC}_{0-\infty }$ to reduce uncertainty from terminal-phase extrapolation. This approach is appropriate because ${AUC}_{0-t}$ is based on observed concentration–time data. However, the oral AUC extrapolated was relatively high, indicating greater uncertainty in the terminal-phase estimation after oral dosing. Therefore, the estimated bioavailability should be regarded as preliminary. Extended sampling designs and additional dose levels would be useful to confirm the extent of oral absorption and to further evaluate the reliability of bioavailability estimates for matrine in pigs.

Another notable observation was that the apparent terminal half-life estimated after oral administration was longer than that after intravenous dosing. However, visual inspection of the concentration–time profiles showed that the terminal portions of the intravenous and oral curves overlapped and appeared approximately parallel. Therefore, the longer apparent half-life after oral administration should not be interpreted as definitive evidence of flip-flop kinetics or slower systemic elimination [28,29]. Under simple linear pharmacokinetic conditions, the terminal elimination half-life is generally expected to be independent of the route of administration [15,16,17,24]. Because the present study used non-compartmental analysis and did not estimate the absorption rate constant (Ka), comparison between Ka and the elimination rate constant could not be performed. The difference in estimated terminal half-life may be partly related to uncertainty in terminal slope estimation, the limited 24 h sampling duration, variability in terminal concentration points, and possible differences in the data points selected for λz estimation [21,22,30]. Therefore, the oral terminal half-life should be interpreted cautiously as an apparent parameter. Further studies with extended sampling, compartmental modeling, or deconvolution analysis are required to more accurately characterize the absorption and elimination phases of matrine in pigs.

Several limitations of this study should be acknowledged. First, the sample size was modest, although it is acceptable for an exploratory pharmacokinetic study in pigs. Second, the present work focused on plasma pharmacokinetics after a single administration and did not assess tissue distribution, metabolite profiles, pharmacodynamic endpoints, repeated-dose exposure, or dose proportionality. Third, although both male and female pigs were included to improve biological representativeness, the study was not designed or powered to assess sex-related pharmacokinetic differences. After stratification by both sex and administration route, the number of animals in each subgroup would be insufficient for reliable statistical comparison. Fourth, AUC extrapolated after oral administration exceeded 20%, indicating that the oral terminal phase was insufficiently characterized; therefore, oral ${AUC}_{0-\infty }$, λz-derived T_1/2_, CL/F, Vz/F, and absolute oral bioavailability were not reported or interpreted. Finally, safety assessment was limited to routine clinical observation, and no systematic hematological, biochemical, or physiological monitoring was performed. Therefore, future studies with larger sample sizes, extended sampling, tissue distribution analysis, pharmacodynamic integration, and repeated-dose safety evaluation are warranted.

## 5. Conclusions

In conclusion, this study characterized the plasma pharmacokinetics of matrine in pigs after intravenous and oral administration and provided a preliminary estimate of its absolute oral bioavailability. Because the percentage of extrapolated AUC after oral administration exceeded 20%, terminal-phase-dependent oral parameters, including CL/F, Vz/F, T1/2, and absolute oral bioavailability, were not reported. These findings provide a pharmacokinetic basis for future PK/PD-guided dose optimization of matrine in swine. Nevertheless, definitive dosing regimens require further studies incorporating pharmacodynamic targets, tissue distribution, repeated-dose exposure, efficacy, and safety evaluation.

## Figures and Tables

**Table 1:** Pharmacokinetic parameters of matrine in pigs following single intravenous and oral administration at 8 mg/kg body weight.

Parameter (Units)	Route of Administration	
	i.v. (*n* = 6)	p.o. (*n* = 6)
${AUC}_{0-t}$ (h·ng/mL)	558.01 ± 59.57	418.94 ± 75.52
${AUC}_{0-\infty }$ (h·ng/mL)	634.58 ± 67.91	NR
AUC extrapolated (%)	11.98 ± 4.19	38.24 ± 14.40
λz (1/h)	0.176 ± 0.039	NR
CL or CL/F (mL/h)	16,958.62 ± 1876.93	-
MRT_0−t_ (h)	2.72 ± 0.58	7.36 ± 1.70
V_z_ or V_z_/F (mL)	99,094.76 ± 17,708.86	-
$C_{max}$ (ng/mL)	224.64 ± 20.94	66.24 ± 8.44
$T_{max}$ (h)	-	2.49 ± 0.02
T_1/2_ (h)	4.09 ± 0.8	NR

Abbreviations: ${AUC}_{0-t}$, area under the plasma concentration–time curve from time 0 to the last measurable concentration; ${AUC}_{0-\infty }$, area under the plasma concentration–time curve from time 0 extrapolated to infinity; AUC extrapolated, percentage of AUC extrapolated from the last measurable concentration to infinity; λz, terminal elimination rate constant; $C_{max}$, maximum observed plasma concentration; $T_{max}$, time to maximum observed plasma concentration; T_1/2λz_, terminal elimination half-life; MRT_0−t_, mean residence time from time 0 to the last measurable concentration; CL, systemic clearance after intravenous administration; Vz, apparent volume of distribution during the terminal phase after intravenous administration; NR, not reported. For the oral group, AUC0-infinity, λz-derived T_1/2_, CL/F, Vz/F, and absolute oral bioavailability were not reported because AUC extrapolated exceeded 20%, indicating insufficient reliability of terminal-phase estimation. Data are presented as mean ± standard deviation (SD). SD, standard deviation.

## Data Availability

The data presented in this study are available on request from the corresponding author. (The data from this study are currently being used as part of a new veterinary drug registration application, and the application is still under review. For this reason, it is not convenient for us to disclose the minimal dataset further at this stage).
